# Diffuse correlation spectroscopy blood flow monitoring for intraventricular hemorrhage vulnerability in extremely low gestational age newborns

**DOI:** 10.1038/s41598-022-16499-3

**Published:** 2022-07-27

**Authors:** John Sunwoo, Alexander I. Zavriyev, Kutlu Kaya, Alyssa Martin, Chelsea Munster, Tina Steele, Deborah Cuddyer, Yvonne Sheldon, Felipe Orihuela-Espina, Emily M. Herzberg, Terrie Inder, Maria Angela Franceschini, Mohamed El-Dib

**Affiliations:** 1 Athinoula A. Martinos Center for Biomedical Imaging, Massachusetts General Hospital, Harvard Medical School, Boston, MA USA; 2 Department of Pediatric Newborn Medicine, Brigham and Women’s Hospital, Harvard Medical School, Boston, MA USA; 3 School of Computer Science, University of Birmingham, Birmingham, UK; 4 Division of Neonatology and Newborn Medicine, Department of Pediatrics, Massachusetts General Hospital, Harvard Medical School, Boston, MA USA

**Keywords:** Paediatric research, Biomedical engineering, Predictive markers, Optical spectroscopy

## Abstract

In premature infants with an extremely low gestational age (ELGA, < 29 weeks GA), dysregulated changes in cerebral blood flow (CBF) are among the major pathogenic factors leading to germinal matrix/intraventricular hemorrhage (GM/IVH). Continuous monitoring of CBF can guide interventions to minimize the risk of brain injury, but there are no clinically standard techniques or tools for its measurement. We report the feasibility of the continuous monitoring of CBF, including measures of autoregulation, via diffuse correlation spectroscopy (DCS) in ELGA infants using CBF variability and correlation with scalp blood flow (SBF, served as a surrogate measure of systemic perturbations). In nineteen ELGA infants (with 9 cases of GM/IVH) monitored for 6–24 h between days 2–5 of life, we found a strong correlation between CBF and SBF in severe IVH (Grade III or IV) and IVH diagnosed within 72 h of life, while CBF variability alone was not associated with IVH. The proposed method is potentially useful at the bedside for the prompt assessment of cerebral autoregulation and early identification of infants vulnerable to GM/IVH.

## Introduction

Approximately 1–2% of all births worldwide occur at the extremes of immaturity (extremely low gestational age, ELGA, < 29 weeks GA), resulting in approximately 60,000 affected newborn infants in the United States each year^1^. Of these, approximately 15,000 (25%) are diagnosed with a germinal matrix/intraventricular hemorrhage (GM/IVH) that often results in adverse neurodevelopmental outcomes or death^2–4^. Moreover, 90% of the GM/IVH cases are diagnosed within the first 72 h after the birth, highlighting the importance of close monitoring during this critical period^2,5^.

One proposed etiology of GM/IVH is poor regulation of cerebral blood flow (CBF) contributing to frequent episodes of: (1) cerebral hypo-perfusion followed by hyper-perfusion and/or (2) passive CBF changes to systemic influences, such as blood pressure (BP). These episodes are related to an impaired cerebral autoregulation (CAR), resulting in a direct injury to the GM, which has a fragile, dense vasculature that remains underdeveloped until 33 weeks of gestation^2,4,6–9^. Therefore, continuous monitoring of CBF and its autoregulation in ELGA infants may guide practices to reduce the risk of brain injury, including GM/IVH, especially during the period of physiological instability in the first 72 h of life.

Diffuse correlation spectroscopy (DCS) is a non-invasive, optical CBF monitoring technique that can be utilized at the bedside for continuous monitoring. DCS utilizes near-infrared light and provides an index of cerebral blood flow (CBF_i_, cm^2^/s) by quantifying the temporal fluctuations of coherent light scattered by moving red blood cells^10,11^. DCS has been validated with the relative and absolute changes in cerebral blood perfusion obtained by ‘gold standard’ methods in humans^12^ and animals^13–15^.

Previous studies have shown the potential clinical utility of DCS-based CBF analysis in extremely preterm infants, with the reports of distinctive fluctuating patterns in CBF between days 1 and 3 of life^16^ and significantly reduced CBF in the presence of low-grade IVH^8^. However, additional research is required to evaluate the clinical utility of DCS monitoring in the context of impaired CAR and IVH vulnerability in ELGA infants.

Meanwhile, signal correlation-based indices of CAR have previously been measured in studies demonstrating that a strong correlation (or coherence) between cerebral hemodynamics and BP was associated with brain injury and IVH^17–24^; however, these studies relied on surrogate measures of CBF with invasive arterial lines for BP (e.g., Near-infrared spectroscopy-based cerebral oximetry or transcranial Doppler velocity). This makes the measurement and interpretation of CAR more difficult, as cerebral oximetry does not provide a direct measurement of blood flow^25^ and the interpretation of the data can be controversial^8,26,27^. In addition, transcranial Doppler velocity cannot be used continuously and can only provide few snapshots of CAR. Furthermore, arterial BP monitoring is not used on all ELGA infants due to its invasiveness, while the cuff-based non-invasive alternative does not work well in small infants^28^.

To address these challenges, we proposed DCS-based CBF variability and correlation indices to quantify the loss of CAR, by means of [coefficients of variation (CV) in CBF] and [cross-correlation coefficients between CBF and scalp blood flow (SBF, which served as a surrogate measure of BP and other systemic perturbations)], respectively. And we evaluated the quantified indices of CAR in association with the development of GM/IVH in ELGA infants.

## Methods

### Subjects and setup

This study was reviewed and approved by the Mass General Brigham Human Research Committee (Institutional review board #2014P002022; approved January 27, 2015) and was conducted at Brigham and Women’s Hospital. ELGA infants born < 29 weeks GA, < 48 h of life, with no major congenital anomalies or complex cyanotic congenital cardiac disease, were enrolled in the study. The study was explained to the parents of an eligible baby by a study staff nurse or physician, and the informed consent was obtained. Once enrolled, each infant was monitored with DCS for ~ 3–6 h a day for up to 3 consecutive days. All methods were performed in accordance with the relevant guidelines and regulations.

**Figure 1:**
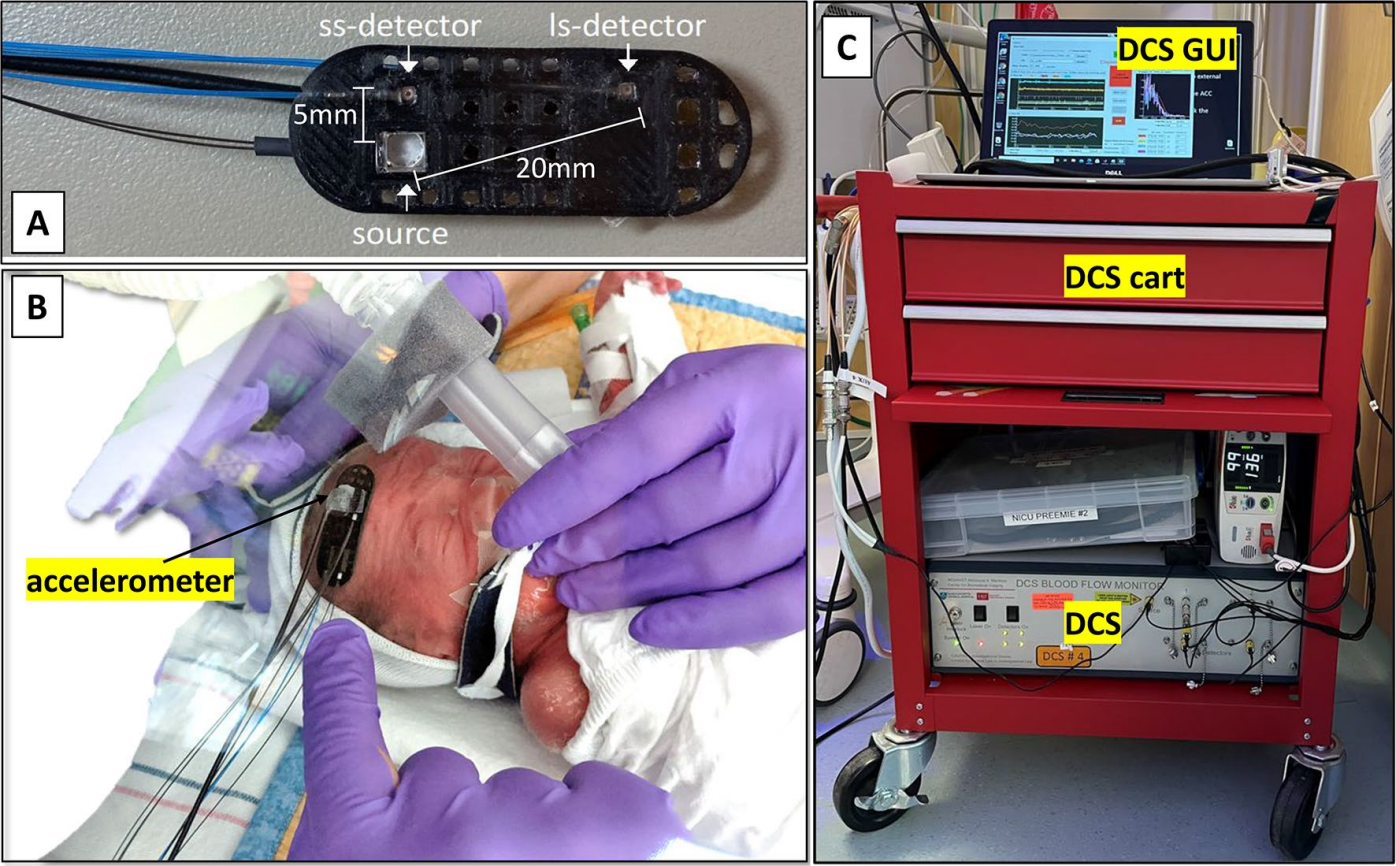
The DCS optical sensor included 5 mm/short- and 20 mm/long-separation channels (**A**) and an accelerometer (**B**). The sensor was attached to the infant’s head with a hydrogel to ensure skin integrity during the long monitoring sessions and was covered by a continuous positive airway pressure (CPAP) hat (**B**). The DCS cart was equipped with a DCS device and a computer running the DCS graphical user interface (**C**). Arterial blood pressure measurements, when available, were co-recorded through an auxiliary port on the DCS device. (For B, the informed consent was obtained from the parent for publication of identifying images in an online open-access publication.)

A custom, flexible and lightweight, optical probe connected to a home-built DCS device^29^ was placed on the forehead as shown in Fig. 1 and kept in place with a thin hydrogel cutout, sandwiched between the probe and the skin. Source-detector separations of 5 and 20 mm were used to acquire scalp and brain blood flow, respectively. In addition, electrocardiogram (EKG) and, when available, arterial BP data (Carescape Monitor B850, General Electric) were co-recorded through auxiliary input ports on the DCS device.

The study operator surveilling a video of the infant had the ability to remotely shut down the laser in case of unexpected probe detachment. An accelerometer was attached to the optical probe to alert the operator about head movements and its signal was used in post-processing to reject segments of data containing motion artifacts.

### IVH diagnosis

IVH was identified using head ultrasound (HUS) on days 1, 3, 7, and 30 of life, as a part of clinical care. HUS images were first read by a radiologist for the initial impression and were then independently verified by two experienced neonatologists using the criteria for classifying IVH proposed by Mohammad et al.^30^; defined as IVH grade I for a hemorrhage confined to the germinal matrix, grade II for a hemorrhage in the lateral ventricles with no dilation, grade III for a hemorrhage causing acute ventricular dilation (with the anterior horn width > 6 mm), and grade IV for a periventricular hemorrhagic infarction in the presence of GM/IVH.

### DCS device and data processing

The continues-wave DCS device used in the study was built in-house with a long coherence length laser source at 785 nm (DL785-120-S, CrystaLaser), four single-photon avalanche diode detectors (Excelitas Technologies, Canada), one collecting light at the short separation and three at the long separation, and a custom electronics board for the time tagging of the detected/arrived photons. It provided data sampling rates of 150 MHz and 50 kHz for optical and auxiliary data, respectively. The optical data consisted of photon arrival time-stamps, which were used for the computation of the temporal autocorrelation function (g_2_), and subsequent fitting with the correlation diffusion equation using the assumed absorption (*µ*_a_ = 0.1 cm^−1^) and reduced scattering (*µ*_s_′ = 4.0 cm^−1^) coefficients to obtain an index of blood flow (BF_i_)^31,32^. The principle is based on the changes in temporal autocorrelation decay, in response to varying speeds of moving red blood cells in the tissue. We developed a custom graphical user interface to receive photon packets with time-stamps, process them into temporal autocorrelation functions and BFi, and display them alongside auxiliary signals in real-time. More details of the DCS device and method have been previously published^29^.

Using MATLAB (ver. R2019b, Mathworks, Inc.), the photon arrival time-stamps from each detector were processed into g_2_ decay functions at 1 Hz for convenience. The resulting g_2_ functions were further averaged to fit for BF_i_ at both short and long separations with 5-s resolution (i.e., SBF_i_ and CBF_i_ at 0.2 Hz). This helped to average out cardiac noise and achieve a better signal-to-noise ratio while maintaining necessary temporal resolution. The signal was smoothened by 3 sample points (= 15 s) sliding window averaging using the *smooth* function from MATLAB. In addition, when arterial BP was available, mean arterial pressure (MAP) was computed by $\frac{({Pressure}_{systolic}+{2\times Pressure}_{diastolic})}{3}$ and was also resampled to 5-s resolution.

The on-probe accelerometer signal was resampled at 1 Hz and used for rejection of motion artifacts, by using a sliding standard deviation (SD) window of 30 s and, for any data period that exceeded SD > 0.01 V (equivalent to 0.23 m/s^2^, Analog Devices ADXL327), we rejected the corresponding DCS data. If the accelerometer measurement was not available, EKG signal resampled at 1 Hz followed by the Z-transformation with the threshold at moving SD > 1 (which we empirically found was able to detect similar motion artifacts as the accelerometer) was used for the same motion artifact rejection routine. The 1 Hz downsampling helped to remove the PQRST waves, while maintaining signal fluctuations and baseline drifts due to large movements that changed the sensor contact of EKG electrodes, providing similar information as in an accelerometer. In addition, to remove noisy data and ensure good data quality, we established the following rejection criteria based on empirical assessment: less than 1 h/day of usable data; 20 s around the periods of brief laser off; 1-h blocks with noisy g_2_ functions defined by SD of beta > 0.04, where beta corresponds to the y-intercept of the fitted correlation diffusion equation onto g_2_; The periods of beta outside of the 0.27–0.57 range using a 90-s sliding average window; The periods that had a large distortion in g_2_ functions due to the interference by other optical monitoring devices, like a bedside cerebral oximeter.

### CBF fluctuation index (CV_CBFi_)

**Figure 2:**
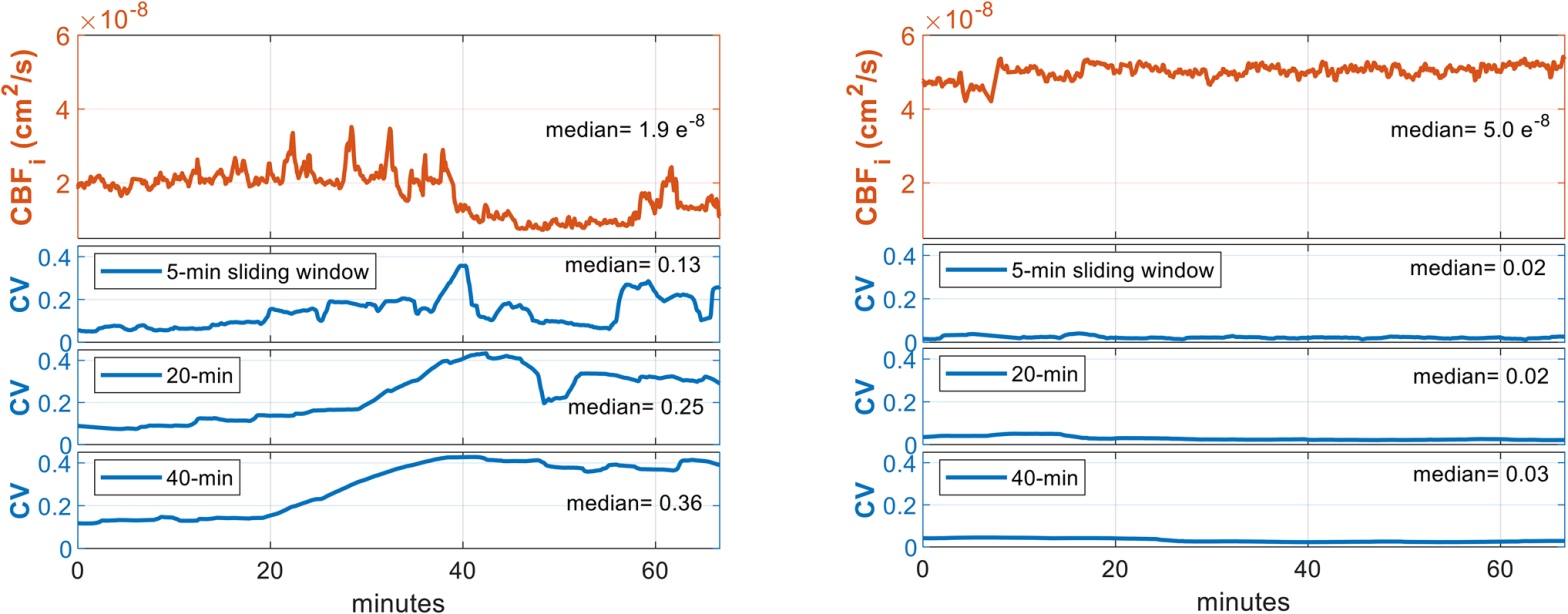
High CBF fluctuation from a 25-week GA ELGA infant with grade IV IVH (Left) was indicated by large (based on the population range shown in Fig. 4b) values of the coefficient of variation (CV) calculated using 5-, 20-, and 40-min sliding windows. Right: In contrast, another ELGA infant with No-IVH (28 weeks GA) showed higher perfusion with low fluctuation, captured by the CV method.

In addition to absolute CBF_i_ daily values, we quantified the variability in CBF_i_ (CV_CBFi_) by computing sliding-window coefficients of variation averaged per day in order to capture the sudden blood flow changes in the brain, normalized by the basal level of CBF. In order to capture different frequency components of CBF fluctuation, CV indices were computed using 5-, 20-, and 40-min sliding windows. The periods with not enough data points (< 75% of the window size) due to the data rejection criteria described above, were excluded from the analysis. Signal examples shown in Fig. 2a illustrate higher CV_CBFi_ values in an infant diagnosed with grade IV IVH, while lower CV_CBFi_ was seen in another infant with no IVH (Fig. 2b). The variability in SBF_i_ and MAP were computed the same way using a 5-min sliding window (CV_SBFi_ and CV_MAP_).

### RiSC: average sliding-window R index between scalp and cerebral blood flow

**Figure 3:**
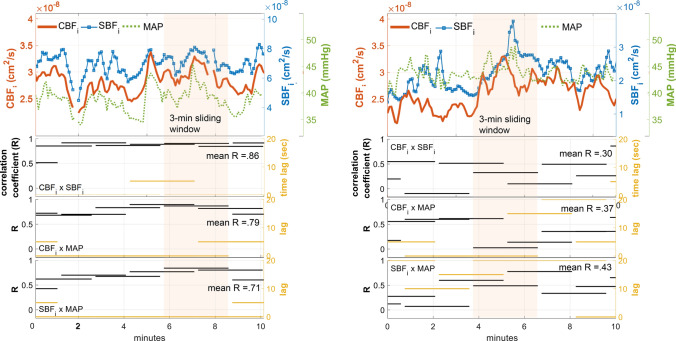
Correlation-based indices of impaired (left) vs healthy (right) cerebral autoregulation showed a possible association with IVH. Left: A 24 week GA ELGA infant with grade IV IVH diagnosed on the same day of the DCS measurement (day 3 of life), showing strong and frequent pressure passive events as the correlation was 0.8–0.9, and the time lags were mostly at 0–5 s between CBF, SBF, and MAP. Right: In an infant with no IVH, CBF (red tracing) was out of phase compared to SBF (blue tracing with square markers) within the 3-min window, as well as in MAP (dotted green tracing) vs CBF (red tracing). On average, the correlation was lower (< 0.5) and with a longer lag (0–20 s) than in the IVH example. For both left and right: Visually, SBF (blue tracing with square markers) showed the signal peaks that were correlated with MAP (dotted green tracing); A 3-min, 50% overlapping sliding window was used to compute the best positive correlation between leading and following signals up to 20 s of time lag; Shown here is a segment of 10 min of data.

To further quantify the loss of CAR, we obtained the daily average correlation between SBF_i_ and CBF_i_. Typically, the signal correlation between MAP and CBF_i_ is considered a marker of impaired cerebral autoregulation, which corresponds to pressure passiveness (Panel row 3 in Fig. 3a). However, continuous BP was not available in more than 50% of the measured infants. Instead, in all infants, we used SBF_i,_ acquired with DCS at a source-detector separation of 5 mm, as a surrogate measure of systemic changes and we performed the same correlation analysis as the pressure passivity analysis. We postulated that scalp blood flow was less tightly autoregulated compared to cerebral blood flow, and was likely to be independent of the changes in cerebral blood flow when CAR is intact. Other external parameters that could affect scalp blood flow, like the changes in ambient temperature, were kept as minimal as possible in a controlled environment (i.e., isolette) and at a much slower rate than the systemic blood pressure changes in ELGA infants^33^. Moreover, in this population, the blood flow measured at large separations is minimally impacted by scalp blood flow, because of the very thin scalp-skull layer in newborn ELGA infants (< 6 mm in preterm infants vs ~ 15 mm in adults)^32,34,35^.

In this study, the best lagged positive correlation between SBF_i_ and CBF_i_ was identified with an assumption that SBF changes would lead to CBF responses. A 3-min, 50% overlapping correlation window was used to detect the signal fluctuation related to pressure autoregulatory vasoreactivity as reported in Lee et al.^36^, and the maximum allowed lag (response) was set to 20 s. Correlation windows with more than 50% of missing data points were excluded from the analysis. Spearman’s rank correlation was used, because we could not assume the linearity between the changes in CBF and SBF, and it could minimize the possible bias from abrupt noise caused by unknown sources^37^. As a result, average response times (T_R_) and the corresponding correlation coefficients (R) were found for each day of the measurements (Fig. 3).

Finally, the daily average of correlation coefficients was obtained and named: RiSC (average sliding-window R index between Scalp and Cerebral blood flow). In addition, T_RiSC_ (average T_R_ associated with RiSC) was computed. Thus, for an infant, a high RiSC score would translate to a higher correlation between scalp and cerebral blood flow, indicating the loss of cerebral buffering of the changes in systemic blood pressure or perfusion.

### Statistical tests

For group analysis, we compared the quantified indices of CAR (e.g., CV_CBFi_ and RiSC) as an outcome of two different aspects of IVH. The first aspect was severity of IVH, (defined as the worst IVH grade of the right and left cerebral hemispheres) in three groups of no-IVH, mild-IVH (grade I or II), and severe-IVH (grade III or IV). The second aspect of IVH was time of IVH onset, and we determined whether the CAR indices were different between the early IVH group, diagnosed within 72 h of life (HoL), versus the rest combined (no-IVH + IVH diagnosed after 72 HoL).

A linear mixed model was used to account for repeated measurements (across different days in each infant) as a ‘random’ effect, and the IVH groups were set as a ‘fixed’ effect. The normality assumption was assessed using normal Q-Q plots and an Anderson–Darling test for each group. In the case of covariates with a small sample size in one group (< 5), the normality assumption was assessed for all of the groups combined (see Supplementary Table S1), followed by the normality assessment of the model residual. The linear mixed model was tested with one covariate at a time for possible confounding variables and interactions. We considered the following factors as possible covariates: GA, bodyweight, sex, Apgar scores, patent ductus arteriosus (PDA), MAP level, CV_MAP_, daily hematocrit (HCT) minimum, the mode of ventilation, and the administration of caffeine, inotropes, indomethacin, or acetaminophen, as listed in Tables 1 and 2. The α level was set to 0.05. All analyses were run using JMP Pro v15 Software (SAS Institute Inc.).

**Table 1:** Demographics and clinical characteristics among N = 19 infants by IVH status.

Variables	Total (N = 19)	No IVH (N = 10)	IVH grade I or II (N = 4)	IVH grade III or IV (N = 5)	*P* value*
Gestational age (weeks)	26.1 ± 1.5	26.1 ± 1.2	26.6 ± 2.1	25.6 ± 1.6	0.61
Birth weight (g)	818.6 ± 175.7	830.9 ± 167.5	891.3 ± 260.7	736.0 ± 102.9	0.42
Apgar at 1 min	3.0 (2.0–5.0)	4.0 (2.0–5.5)	4.0 (3.0–5.0)	2.0 (1.0–2.5)	**0.03**
Apgar at 5 min	6.0 (5.0–7.0)	7.0 (6.0–8.0)	6.0 (5.3–6.0)	5.0 (3.5–6.5)	**0.04**
Male	13 (68.4%)	6 (60.0%)	3 (75.0%)	4 (80.0%)	0.82
Death	4 (21.1%)	0 (0%)	1 (25.0%)	3 (60.0%)	**0.03**
Time of IVH found by HUS (HoL)	–	–	131.1 ± 74.0	44.9 ± 21.4	**0.04**
Time of the 1st DCS measurement (HoL)	31.3 ± 13.4	27.8 ± 11.3	41.5 ± 14.4	30.0 ± 15.1	0.23
Arterial line	8 (42.1%)	5 (50.0%)	1 (25.0%)	2 (40.0%)	0.83
**Delivery**					0.94
C-section	11 (57.9%)	6 (60.0%)	2 (50.0%)	3 (60.0%)	
SVD	8 (42.1%)	4 (40.0%)	2 (50.0%)	2 (40.0%)^b^	
Maternal age (years)	30.6 ± 4.7	31.3 ± 3.9	26.3 ± 6.2	32.6 ± 3.2	0.09
**Length of stay (days)**					
Missing^a^	8 (42%)	3 (30%)	2 (50%)	3 (60%)	0.49
Median (25–75th)	121 (112–155)	120 (112–168)	136 (134–138)	106 (91–121)	0.40
PDA diagnosed by echo	12 (63.2%)	5 (50%)	4 (100%)	3 (60%)	0.32
Bronchopulmonary dysplasia (chronic lung disease)	8 (50%), 3 missing	6 (75%)	2 (50%)	1 (25%)	0.29

Significant values are in bold.

*Linear regression was used for continuous variables that passed the normality test (otherwise Wilcoxon/Kruskal–Wallis Rank Sums test was used) and Fisher’s Exact Test was used for categorical variables; ^a^Missing includes the total days less than 30 (due to early death or a transfer) identified as outliers; ^b^One case of vaginal-breech delivery. *HUS* Head ultrasound, *HoL* Hours of Life, *SVD* Spontaneous Vaginal Delivery. Data are presented as N (Column %) for categorical variables, except when presented as mean ± SD and when presented as median (25–75th).

**Table 2:** Daily clinical variables during the studies among N = 49 days by IVH status.

Variables	Total (N = 49 days)	No IVH (N = 27)	IVH grade I or II (N = 9)	IVH grade III or IV (N = 13)	*P* value*
**MAP (median level/day, mmHg)**					
Available	17 (35%)	11 (41%)	2 (22%)^a^	4 (31%)	0.66
Mean ± SD	36.7 ± 5.5	36.9 ± 3.6	26.4 ± 5.1	41.2 ± 3.6	0.06
MAP coefficient of variation (5-min sliding window median/day)	0.034 ± 0.010	0.036 ± 0.009	0.022 ± 0.006	0.035 ± 0.012	0.42
**HCT (%, daily minimum)**					
Available	34 (69%)	18 (67%)	6 (67%)	10 (77%)	0.84
Median (25–75th)	39.5 (34–41)	39.8 (35–41)	42.0 (40–46)	35.3 (28–39)	0.45^c^
Caffeine^b^	14 (28.6%)	10 (37.0%)	2 (22.2%)	2 (15.4%)	0.36
Indomethacin (for IVH prevention purpose and PDA)^b^	4 (8%)	3 (11%)	0 (0%)	1 (7%)	0.81
Inotropes^b^	6 (12.2%)	4 (14.8%)	2 (22.2%)	0 (0%)	0.19
Acetaminophen^b^	2 (4%)	1 (4%)	1 (11%)	0 (0%)	0.41
**Mode of ventilation (worst/day)**					0.99
Conventional including HFV	25 (51%)	14 (52%)	4 (44%)	7 (54%)	
Non-invasive including CPAP and NIPPV	24 (49%)	13 (48%)	5 (56%)	6 (46%)	

^a^The two measurements were from one subject; ^b^Yes, if administered during the DCS measurement and within 1 h prior to the DCS measurement; *P values were obtained using a linear mixed model (when possible) to account for repeated measures in each subject. For categorical variables, the P-values were obtained using Fisher’s exact test without accounting for repeated measures in each subject.; ^c^Square() transformation was applied. *HCT* Hematocrit percentage in the blood, *CPAP* continuous positive airway pressure ventilation, *HFV* high frequency (oscillation or jet) ventilation, *NIPPV* non-invasive positive pressure ventilation. Data are presented as n (column %) for categorical variables, except when presented as a mean ± SD for normally distributed variables or when presented as a median (25–75th) for non-normal variables.

## Results

### Overview of demographic, clinical, and DCS variables

We enrolled twenty-one ELGA infants between August 2019 and June of 2021 (Tables 1, 2, 3, and 4). Among them, two infants were excluded from further analysis: one infant was excluded due to excessive signal interference by another optical monitoring device and missing EKG and accelerometer measurements required for motion artifact rejection. Another infant, who was measured for only one day, was excluded due to noisy CBF_i_ determined by the beta criteria (see “Methods” section for the beta criteria above). Among the final nineteen ELGA infants, there was one infant measured without an accelerometer and another infant with partially missing accelerometer data (ID #1 measured across two days and ID #14 missing 3 h on day #2, respectively). The mean weight and GA at birth was 819 (± 176) grams and 26.1 (± 1.5) weeks, respectively. Nine infants (47% of total) were diagnosed with IVH by 7 days of life. Of these, six infants (67% of the IVH group) were diagnosed with IVH within 3 days of life. The first DCS measurement was normally conducted on the second day of life (~ 31 HoL) for a duration of ~ 4.3 h a day and this protocol was repeated in each infant, resulting in 52 total measurement days across all infants. Among the measurement days, three were rejected due to noisy CBF_i_ determined by the beta criteria (2) or due to operator error (1).

**Table 3:** DCS variables during the studies among N = 49 days by IVH status.

Variables	Total (N = 49 days)	No IVH (N = 27)	IVH grade I or II (N = 9)	IVH grade III or IV (N = 13)	*P* val*
Duration of the measurement (h/day)	4.3 (3.4–5.8)	3.7 (3.0–5.8)	5.0 (4.1–5.8)	5.0 (3.6–6.2)	0.27^a^
Total data length after MA rejection (h/day)	3.9 ± 1.4	3.6 ± 1.5	4.2 ± 1.2	4.3 ± 1.4	0.33
Motion artifact (%/day)	9.6 (4.9–15.2)	7.4 (4.5–19.8)	11.8 (9.6–20.4)	7.8 (2.9–12.7)	0.28^a^
SBF_i_^d^ (daily median, e-8 * cm^2^/s/day)	5.1 (3.1–6.7)	5.1 (3.2–6.2)	7.0 (4.3–9.4)	3.7 (2.7–5.4)	**0.04**
CBF_i_ (daily median, e-8 * cm^2^/s/day)	3.2 (2.7–4.4)	3.6 (2.8–4.7)	3.0 (2.8–4.1)	2.7 (2.1–3.4)	0.41^a^
CV_SBFi_ (SBF_i_ coefficient of variation) (5-min sliding window median, /day)	0.107 (0.09–0.13)	0.124 (0.10–0.15)	0.107 (0.09–0.12)	0.096 (0.08–0.12)	0.76^b^
CV_CBFi_ (5-min sliding window median, /day)	0.069 ± 0.018	0.070 ± 0.019	0.069 ± 0.020	0.067 ± 0.015	0.95
CV_CBFi_20_ (20-min sliding window median, /day)	0.089 ± 0.022	0.090 ± 0.025	0.088 ± 0.021	0.085 ± 0.015	0.95
CV_CBFi_40_ (40-min sliding window median, /day)	0.101 ± 0.027	0.105 ± 0.032	0.100 ± 0.021	0.094 ± 0.016	0.77
RiSC (3-min sliding window mean, /day)	0.56 ± 0.14	0.53 ± 0.14	0.49 ± 0.12	0.65 ± 0.12	0.17
T_RiSC_ (s/day)	2.8 (1.5–4.5)	3.1 (1.5–4.8)	3.7 (2.8–4.4)	2.5 (1.5–3.3)	0.33^c^
**Pressure passiveness indices**					
Available	17 (35%)	11 (41%)	2 (22%)^e^	4 (31%)	0.66
MAP × CBF cross-correlation (3-min sliding window mean, /day)	0.35 ± 0.17	0.30 ± 0.11	0.22 ± 0.11	0.55 ± 0.16	0.14
Delay of MAP × CBF correlation (mean, seconds/day)	6.6 ± 3.4	7.7 ± 3.3	7.4 ± 2.4	3.3 ± 1.7	0.29

Significant values are in bold.

*MA* Motion artifact; *P values were obtained using a linear mixed model (when possible) to account for repeated measures in each subject. The P-values for categorical variables were obtained using Fisher’s exact test without accounting for repeated measures in each subject.; ^a^log_10_() transformation was applied; ^b^logit() transformation was applied; ^c^Square root transformation was applied; ^d^The variable passed the normality test but is presented in median (25–75th) for easy comparison with CBF_i_; ^e^The two measurements were from one subject; Data are presented as n (column %) for categorical variables, except when presented as a mean ± SD for normally distributed variables or when presented as a median (25–75th) for non-normal variables.

**Table 4:** Individual HUS exams and IVH status with respect to our measurement.

ID	IVH grade (at HUS HoL)				Worst IVH grade	1st DCS measurement after IVH onset by HUS (h)
	1st	2nd	3rd	4th		
1	0 (42)	0 (66)	1 (159)	2 (258)	2	− 96
2	0 (26)	0 (74)	0 (169)	0 (1486)	0	NA
3	0 (20)	0 (71)	0 (168)	0 (718)	0	NA
4	0 (18)	4 (60)	4 (86)	4 (108)	4	− 14
5	0 (15)	0 (62)	0 (160)	0 (713)	0	NA
6	0 (21)	0 (45)	0 (165)	0 (334)	0	NA
7	0 (36)	0 (84)	0 (132)	0 (395)	0	NA
8	0 (18)	4 (69)	4 (163)	4 (330)	4	− 47
9	0 (29)	0 (82)	0 (124)	NA	0	NA
10	4 (17)	4 (45)	4 (94)	4 (138)	4	31
11	0 (40)	0 (88)	2 (186)	2 (207)	2	− 142
12	2 (22)	2 (72)	2 (165)	2 (333)	2	7
13	0 (42)	1 (158)	0 (330)	0 (782)	1	− 120
14	0 (33)	0 (77)	0 (149)	0 (341)	0	NA
15	4 (30)	4 (53)	4 (103)	4 (178)	4	− 16
16	0 (28)	0 (47)	0 (169)	0 (349)	0	NA
17	0 (3)	0 (30)	4 (49)	NA	4	− 17
18	0 (18)	0 (66)	0 (162)	NA	0	NA
19	0 (17)	0 (65)	0 (161)	NA	0	NA

*HUS* Head ultrasound, *HoL* Hours of Life.

*NA* not available, not applicable, or not measured.

Each variable listed in Tables 1, 2, and 3 was compared between the three IVH groups. For statistical tests, data transformation was applied as log_10_[median CBF_i_ level], [daily minimum hematocrit, HCT, level]^2^, $\sqrt{{\mathrm{T}}_{\mathrm{RiSC}}}$, and logit[CV_SBFi_] to meet the normality assumption. (See Supplementary Table S1 and S2 for the details of the normality test and transformations applied.)

Univariate tests indicated that neither GA nor BW were different between the groups (*P* = 0.61 or 0.42, respectively). Apgar scores at 1 and 5 min were significantly lower in the severe-IVH group compared to the no-IVH group (Tukey post-hoc comparison, *P* = 0.03 and 0.04, respectively). In addition, the mortality rate was the highest in the severe-IVH group (*P* = 0.03). Moreover, the average time to IVH onset from birth (determined by head ultrasound, HUS) was significantly earlier in the severe-IVH group than in the mild-IVH group (*P* = 0.04).

As shown in Table 2, the daily average MAP level trended the highest in the severe-IVH group, although there were only two measurements from one subject in the mild-IVH group (*P* = 0.06 for between all groups, *P* = 0.05 between severe and mild-IVH groups using Tukey post-hoc test). The MAP fluctuation did not differ between the groups, as shown by the CV_MAP_ (*P* = 0.42). Administration of medication (i.e., caffeine, indomethacin, inotropes, or acetaminophen) during the measurement was not notably high nor statistically different between the groups.

### No significant association was found between CBF fluctuation and IVH outcomes

**Figure 4:**
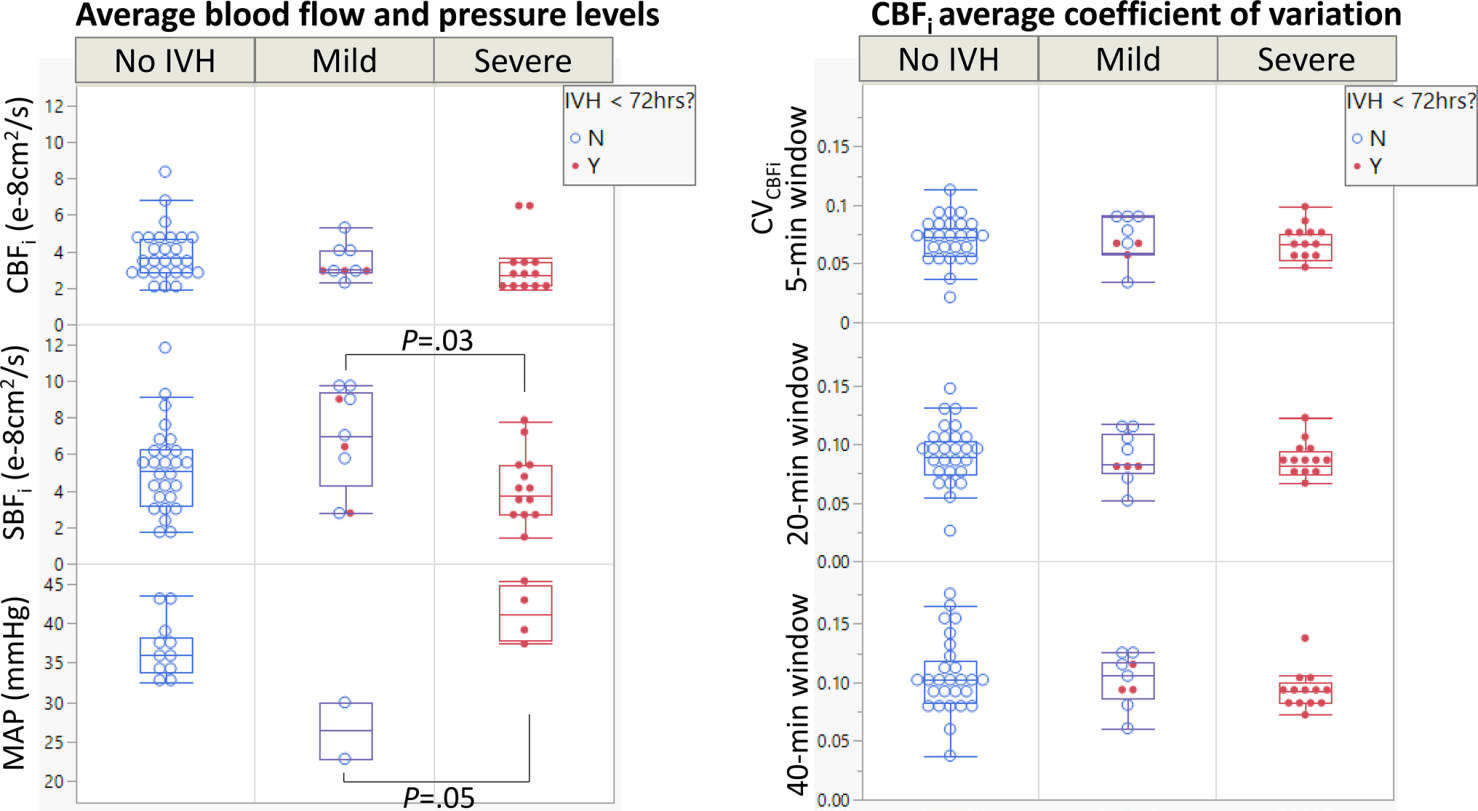
CBF_i_ fluctuation was not different between IVH groups, based on the coefficient of variation method (CV_CBFi_). Each point was based on one day of measurement. Notable differences include a significantly lower scalp blood flow, while showing a higher mean arterial pressure, in the severe-IVH group (*P* values shown are based on Tukey post-hoc test. A linear mixed model was used to account for multiple measurements in each subject).

The DCS-based variables and computed indices are shown in Table 3. The average data rejection due to motion artifacts or other external factors was 9.6% in all subjects (7.4, 11.8, and 7.8% in no-, mild-, and severe- IVH groups, respectively). As illustrated in Table 3 and Fig. 4, the SBF_i_ levels differed between the three groups (*P* = 0.04), showing lower SBF_i_ levels in the severe-IVH group compared to the mild-IVH group (Tukey post-hoc *P* = 0.03). In contrast, we did not find a difference in CBF_i_ levels between the groups (*P* = 0.41). We also did not find differences in CBF_i_ fluctuation between the groups (computed by CV_CBFi_ using 5-, 20-, or 40-min windows, *P* = 0.95, 0.95, or 0.77, respectively). Moreover, when we tested CBF_i_ levels and CBF_i_ fluctuations using the [IVH < 72 HoL Yes/No] groups, no differences emerged (CBF_i_ levels *P* = 0.29; CBF_i_ fluctuations using 5-, 20-, or 40-min windows were *P* = 0.54, 0.63, and 0.50, respectively, between the closed red and open blue circles in Fig. 4). Supplementary Table S3 lists the significant covariates, including Apgar score at 5 min (showed a larger fluctuation with greater Apgar, *P*_*Apgar*_ = 0.04), MAP fluctuation (strong positive correlation, *P*_MAP_ < 0.001), and mode of ventilation (a larger fluctuation with non-invasive modes of ventilation, *P*_ventilation_ = 0.04), although they did not change the main effect observed between the IVH groups and CBF_i_ fluctuation. A significant interaction was found, showing a negative correlation between daily minimum HCT^2^ and CBF_i_ fluctuation in the severe-IVH group, while showing a positive correlation between the two variables in the no-IVH and mild-IVH groups (*P*_IVH*HCT^2_ = 0.04, supplementary Table S3).

### Severe-IVH showed the highest RiSC compared to other groups

**Figure 5:**
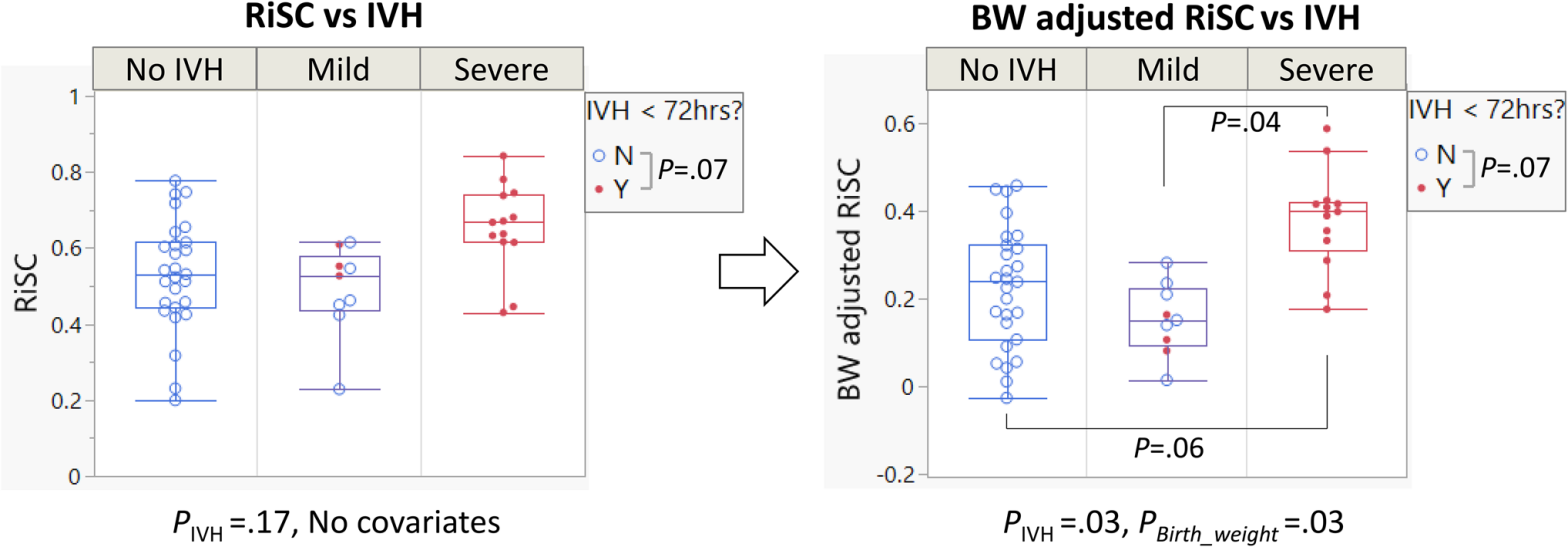
RiSC was higher in the severe-IVH group compared to other IVH groups after accounting for the effect of birthweight (Right panel; Tukey post-hoc test showed *P* = 0.04 in severe vs mild groups and *P* = 0.06 in severe vs no-IVH groups). Each point was based on one day of measurement. The plot on the left shows the original distribution before adjusting for body weight. The right panel shows the RiSC comparison after adjusting for the birthweight. Both the original RiSC and adjusted RiSC were marginally different in ELGA infants who were diagnosed with IVH within 72 h of life compared to controls (*P* = 0.07 and 0.07, respectively). (Red dot = diagnosed with IVH within 72 h of life; Blue open circle = no IVH within 72 h of life.)

We found an association between RiSC indices and IVH after accounting for birthweight (*P* = 0.03, Fig. 5 and Supplementary Fig. S2a). Tukey post-hoc tests showed a higher RiSC in the severe-IVH group compared to the mild-IVH group, and a marginally higher RiSC in the severe-IVH group compared to the no-IVH group (*P* = 0.04 and 0.06, respectively). Before accounting for birthweight, RiSC showed a moderate association with IVH status (*P* = 0.17, Fig. 5, Table 3). Other covariates did not achieve significance and did not show significant interactions with RiSC.

As listed in Table 3, the response times quantified by T_RiSC_ showed a weak trend of shorter response times in severe-IVH (*P* = 0.33). In addition, when we tested the [IVH < 72HoL Yes/No] groups in the same model, the RiSC was marginally higher (*P* = 0.07, open vs. closed circles in Fig. 5) in ELGAs with IVH diagnosis within 72 HoL compared to the control group.

Pressure passivity indices quantified by the cross-correlation of MAP and CBF_i_ also showed a similar contrast between the IVH groups, even with the limited observations, showing the greatest pressure passivity in the severe-IVH group (Table 3). We also compared the indices of RiSC and MAP-based pressure passiveness and found a positive indication of the correlation between the two metrics (Not shown; See Supplementary Fig. S1).

## Discussion

We proposed DCS-based indices of cerebral blood flow and its autoregulation that can be used for continuous risk assessment of GM/IVH in extremely low gestational age infants. The proposed indices were CV_CBFi_ (CBF variability) and RiSC (Cross-correlation indexed by SBF x CBF) that could reflect the loss of cerebral autoregulation in infants with IVH. While we did not find a clear association between CV_CBFi_ and IVH, we found that a greater RiSC was associated with severe IVH, and it was a marginally greater in infants with IVH that occurred within the first 72 h of life.

The higher RiSC observed in the severe-IVH group compared to the no- or mild-IVH cases strongly supports that frequent episodes of CBF dysregulation could lead to severe IVH. Similar observations could be made based on the shorter correlation delay (T_RiSC_) seen in the severe-IVH cases, indicating a greater passivity in CBF to extracerebral influences, although the T_RiSC_ did not achieve statistical significance (Table 3). In any case, RiSC and T_RiSC_ showed a significant correlation with each other (*P* < 0.001, See Supplementary Fig. S2c), suggesting that they may interchangeably serve as a biomarker of cerebral autoregulation function in infants susceptible to IVH.

One of the advantages of our method was that we quantified the best positive correlation within a set response window (up to 20 s), which could directly address the pressure-passiveness when using MAP and CBF measurements. Conversely, an instantaneous correlation of the two signals cannot capture the temporal component of the response to stimuli nor the time-delayed pressure passive events. This can lead to results that are difficult to understand, such as the recent finding of a significant negative correlation between cerebral oxygenation and MAP in infants with IVH, which warrants further explanation^23^.

Another advantage of our method was its ability to provide an index of cerebral autoregulation without the knowledge of arterial pressure obtained from invasive arterial lines in small infants. There are more neonatal clinicians trying to avoid placing invasive arterial lines unless absolutely necessary due to the potential complications. The cuff-based non-invasive alternatives were not feasible in these extremely small infants due to reliable measurement challenges and potential harm to the skin and limb. To address this challenge, we assumed that the scalp blood flow reflected the changes of systemic physiology, such as changes in heart rate and blood pressure. Along the same lines, there are previous reports that have used heart rate as a surrogate measure of systemic changes to derive a cerebral oxygenation reactivity index, which was higher in infants at risk of IVH^38,39^. Furthermore, although it is not clear in the premature infant population, numerous studies in adults have shown that the fast (i.e., 1–2 min) changes in arterial BP are well correlated with changes in superficial blood flow, especially in ‘non-glabrous’ (e.g., forehead and forearm) as opposed to ‘glabrous’ skin (e.g., palm, ear, and sole)^40,41^. Moreover, an abruptly decreased arterial BP caused a decreased flow in the internal carotid artery, which is the main blood supplier to the forehead^42,43^. We assumed that these aspects of physiology were independent of CBF when the brain was properly autoregulating. In our population, although the correlation between SBF_i_ and MAP varied case-by-case (Fig. S2a) and the sample size was small, RiSC showed a significant correlation with the conventional MAP-based pressure passivity indices when we excluded one possible outlier (Fig. S2b). The above information suggested that the MAP could not be replaced by SBF; however, there was necessary information in SBF that reflected the systemic perturbations similar to what MAP provides. Therefore, DCS-based scalp vs brain blood flow monitoring could potentially be used as a non-invasive cerebral autoregulation monitor, at least in the preterm infant population.

Furthermore, our method is well-suited for monitoring preterm infants, as their thin extracerebral layers allow for a better sensitivity to CBF. The influence of cerebrospinal fluid, skull, and scalp thickness is negligible in the long separation DCS measurements (i.e., CBF_i_), as infant skulls are considerably thinner than adults skulls (~ 6 mm vs. ~ 15 mm combined)^32,34,35,44^. Statistical tests showed that adjusting for birthweight led to a significant association between RiSC and IVH status. Meanwhile, there was no relationship between the birthweight and the RiSC alone in all subjects (*P* = 0.51; a linear mixed model was used; The birthweight was categorized into below-average or above-average birthweight groups; Not shown). This indicated that the birthweight alone would not replace the main effect (i.e., IVH status), but served as an important control variable that helped to explain the extra variability in the RiSC; this could be explained by the contribution of head circumference, which was correlated with birthweight, as a smaller head size with thinner scalp, skull, and cerebrospinal fluid could provide better sensitivity to the brain, while a bigger head size could lower the sensitivity, due to the increased extracerebral components into the long separation detectors of the DCS (Head circumference vs birthweight *P* < 0.01; But only 11 measurements were available in our population total of 19 infants at birth). Even so, the possible effect of head size did not appear to be significantly associated with the main effect on the multivariate analysis in this population. This showed that our scalp vs cerebral blood flow correlation method was highly applicable for quantifying the loss of CAR in small infants, owing to a smaller head size.

There are remaining questions. It is difficult to explain the large variation of RiSC in the no-IVH group, making them less distinguishable from the IVH cases. It is possible that the healthier physical condition from birth, indicated by the higher Apgar scores in the no-IVH group, enabled the infants to better sustain and tolerate the pressure passive events and dysregulated cerebral blood flow compared to other infants who were usually born with lower Apgar scores and ended up with IVH (See Supplementary Fig. S3). However, having the Apgar score as a covariate in the model did not help in separating the IVH groups, due to a large range of RiSC values with high Apgar scores (*P* = 0.12 and *P*_*Apgar_5*_ = 0.34; Supplementary Table S3). There were other covariates that we have tested for their influence on our model, but we were often underpowered by the small sample size and unbalanced distribution across the groups, which warrant further investigation as we enroll more subjects (See Supplementary). Another remaining question stands: it is not clear why the absolute scalp blood flow levels were higher than that of cerebral blood flow in our study population, as we typically see the opposite trend in adults (Table 3, CBF_i_ vs SBF_i_ levels in all groups). This may be explained by relatively suppressed CBF in the cortex area during rapid infant development, in contrast with the elevated CBF seen in other regions of the brain (e.g., cerebellum), as has been reported using PET (positron emission tomography) and MRI (magnetic resonance imaging)^45^. And while the infants receive care in a controlled isolette environment, it is possible that elevated metabolic activity during active sleep states may be responsible for the increased SBF^46^.

The study has several limitations. First, the exact timing of IVH onset could not be determined using sporadic HUS exams on days 1, 3, and 7 of life alone, making it more difficult to precisely validate our method. Second, RiSC cannot be validated easily in older children and adults, as their contribution of scalp blood flow in long separation measurements would not be negligible. Third, we are limited by assuming the same optical properties across all infants, as the absolute level of BF_i_ is estimated using the optical properties, and this may be one reason for not finding a difference in the CBF_i_ levels between no-, mild- and severe- IVH groups. However, obtaining optical properties of human tissue is not trivial and usually requires time- and wavelength- resolved near-infrared spectroscopy^47^. Nevertheless, our correlation-based method was independent of absolute BF_i_ levels and was able to provide meaningful trends in CAR to extracerebral fluctuations, in the aspects of severity and time onset of IVH, which could help identify the infants at a higher risk of IVH.

In future work, we will collect more data to further validate our method. For example, we anticipate extending the monitoring time to a continuous 72-h period, which will enable us to acquire and analyze the slow changes in cerebral autoregulation. In addition, we are approved to consent mothers antenatally, which will allow us to monitor infants on the day they are born. With more data points over a continuous 72-h period, we can also validate whether the daily CAR indices can track the development of IVH, as we plot the RiSC over the time distance from [time of the DCS measurement]-to-[time of IVH diagnosed by HUS] in Supplementary Figure S4. In addition, a larger sample size can help develop a more comprehensive model, such as a multiple-input prediction model using RiSC and other covariates as main effects. More measurements with continuous BP can further help understand the relationship between MAP and scalp BF in ELGA infants. Moreover, we will consider collecting and analyzing other important factors that might have perturbed CAR and contributed to IVH, such as sleep versus awake, fluctuations in isolette temperature, and the times of handling and repositioning of the infants. We will also assess the potential benefits of additional post-processing or data transformation, such as Fisher’s Z-transformation to correlation coefficients. Further investigation is needed in CBF variability analysis using advanced algorithms, such as machine learning for pattern recognition.

## Conclusion

In this study, we proposed a novel methodology for assessing GM/IVH risk in ELGA infants using continuous cerebral blood flow monitoring via diffuse correlation spectroscopy, including signal analyses based on blood flow variability and cross-correlation. Our method demonstrated the loss of cerebral autoregulation in severe and early onset IVH cases, indicated by elevated and tightly coupled correlation episodes between scalp blood flow and cerebral blood flow. Ultimately, we believe that our method can help clinicians make better-informed decisions when treating ELGA infants and can foster the development of new interventions to better manage cerebral hemodynamics of premature infants vulnerable to GM/IVH.

## Supplementary Information

Supplementary Information.

## Data Availability

The datasets collected and analysed during the current study are available from the corresponding author on reasonable request.
