# Supplementary material for: Diffuse correlation spectroscopy blood flow monitoring for intraventricular hemorrhage vulnerability in extremely low gestational age newborns
Source: Sci Rep. 2022 Jul 27;12:12798. doi: 10.1038/s41598-022-16499-3 (PMC9329437; doi:10.1038/s41598-022-16499-3)
Supplement: Supplementary file 1 — Supplementary Information. [file 41598_2022_16499_MOESM1_ESM.pdf]

Supplementary

Scalp BF_i as a surrogate measure of MAP and for pressure passivity analysis

We assessed the similarity of Scalp BF_i and MAP signals using a zero-lag, 50% overlapping, 3-minute sliding window cross-correlation, which suggested a positive association between the two signals (P=0.004, one-tailed t-test without adjusting for repeated measures in each subject) (Figure S1). Then, to see if RiSC could be a surrogate index of pressure passiveness, RiSC values were compared with the average correlation coefficient between MAP and CBF_i obtained in all seven available infants with an arterial line (a total of 17 daily measurements). Although there was no significant correlation based on the linear fit and R^2, these two measurements achieved a significant correlation when one possible outlier was excluded (*P*=.31 using all the available observations or *P*=.046 after excluding one outlier point as shown in Figure S1-b).

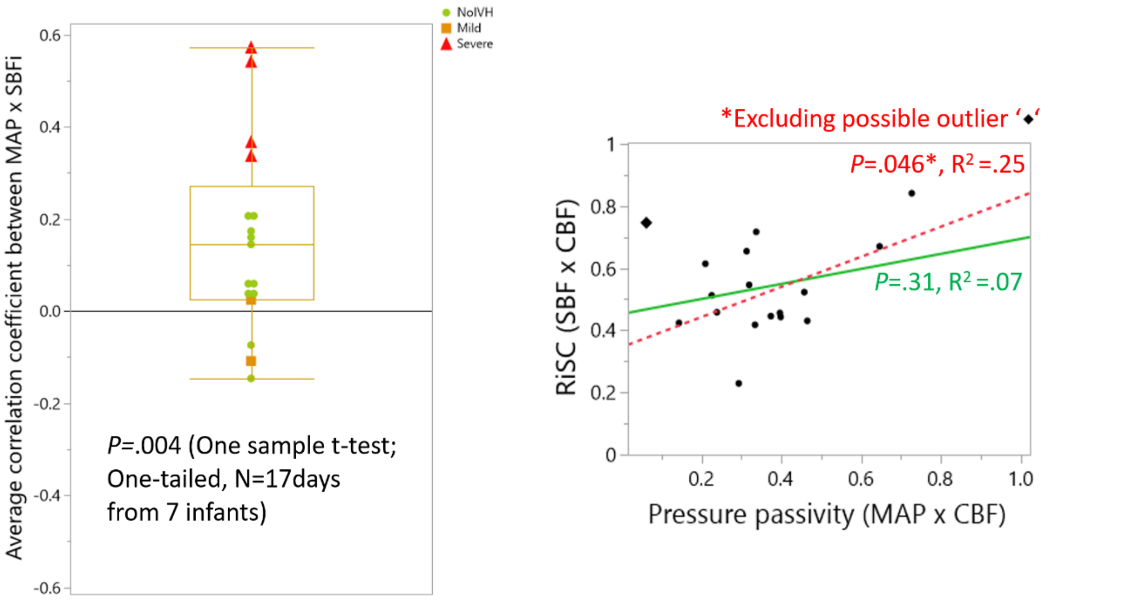

a) Correlation between SBF_i and MAP b) RiSC vs MAP-based pressure passivity indices

Figure S1. Similarity between scalp BF_i and MAP, as well as between RiSC and a conventional index for pressure passiveness, MAP x CBF_i correlation for the 17 available daily measurements in 7 ELGA infants with an arterial line. Each point was based on one day of measurement. a) The average signal correlation between scalp BF and MAP is generally in a positive direction, but this was not always the case. b) The correlation between RiSC and MAP x CBF_i correlation coefficient average suggests the possible utility of RiSC as a biomarker of pressure-passiveness.

Potentially important covariates to be considered in future work

There were other notable covariates in the model (as summarized in Table S3). Apgar score at 5 minutes, by itself, served as a good indicator of IVH vulnerability, and was positively correlated with CV_{CBFi} (*P*=.04, showed a larger CBF_i fluctuation in greater Apgar scores; Figure S2-g). This could indicate an intrinsically greater fluctuation in cerebral perfusion due to the active brain development in healthier infants, but the same fluctuations could be harmful in less-healthy infants, leading to IVH. This suggested a further investigation of the CBF fluctuation analysis in infants after stratifying by poor and normal Apgar scores; however, we did not have enough samples for the stratification. Similarly, there was an interaction between the daily minimum HCT^2 level and the CBF fluctuations of the three IVH groups, as there was a negative correlation between the HCT level and CBF fluctuation in the severe-IVH group, while showing an anticipated, positive correlation between the two variables in other groups, but we were underpowered by the small sample size in the severe-IVH group for further investigation. MAP fluctuation showed a strong positive correlation with the CBF fluctuation in the model (*P*<.001; Figure S2-d), while the administration of caffeine or intubated ventilation may have lowered the CBF fluctuation (*P*=.06 or <0.01, respectively). This could indicate that our infant population had a strong tendency for poor autoregulatory capacity in general, as the magnitude of fluctuation in CBF was highly influenced by the changes in MAP, while clinical interventions suppressed the CBF fluctuation. In addition, this may explain why we did not see a contrast in CBF fluctuation between the groups.

In the case of the RiSC model, besides the effect of birthweight that was discussed in the main text, none of the covariates showed significant effects on RiSC, as summarized in Table S3.

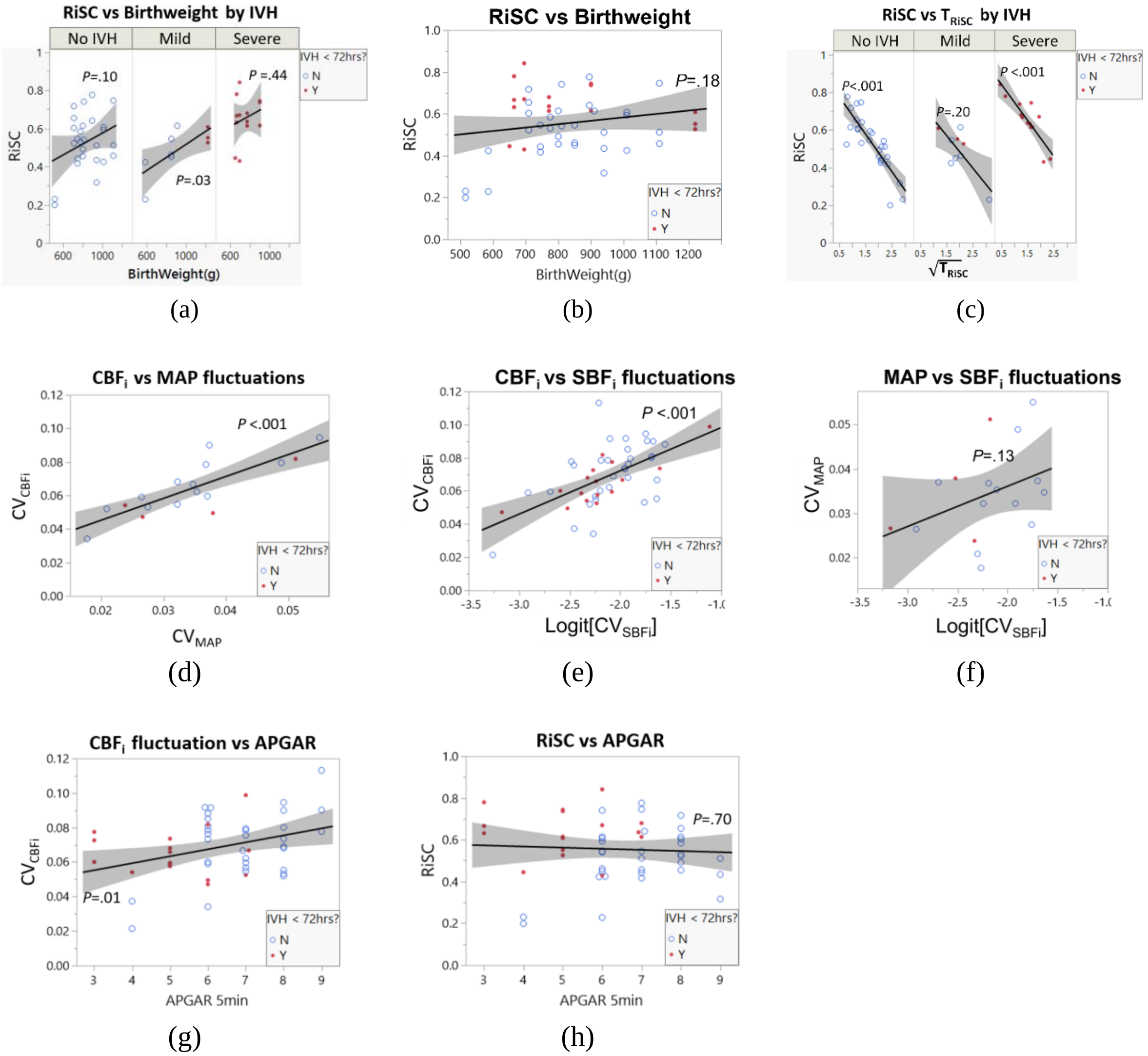

Figure S2. Suggested CAR indices (i.e., CV_{CBFi} and RiSC) in relation with other variables, (a) showing significant birthweight contribution in RiSC when fitted for IVH groups, while birthweight alone did not show a clear association with RiSC (b). The panels (c) show the highly correlated nature of RiSC and T_{RiSC}. Panel (d) showed a strong positive correlation between the fluctuations of CBF_i and MAP, with limited observations in MAP. The fluctuation of SBF_i also showed a strong correlation with the CBF_i (e) and a weakly trended correlation with MAP (f). Panels (g) and (h) show the CAR indices compared to the Apgar 5min score, showing a more clear trend between CBF fluctuation and Apgar score. *P*-values were computed without accounting for the repeated measures in each subject.

Table S1. Normality test and transformation for covariates

**Variables**	**N (available /total possible)**	**Normality test (*P-value)**	**Transformation function**	**Normality after transformation (P-value**
**Gestational age** (weeks)	19/19	.38	-	-
**Birth weight** (g)	19/19	.90	-	-
**Apgar at 1 min**	19/19	.15	-	-
**Apgar at 5 min**	19/19	.28	-	-
**Time of IVH found by HUS** (HoL)	9/9	.07	-	-
**Time of the 1st DCS measurement** (HoL)	19/19	.48	-	-
**Maternal age** (years)	19/19	.30	-	-
**Length of stay** (days)	[L]11/19	.02	$\log_{10}()$	.85
**Duration of DCS measurement** (hr/day)	49/49	.026	$\log_{10}()$	.31
**DCS data length after MA cleaning** (hr/day)	49/49	.08	-	-
**Motion artifact** (%/day)	49/49	<.0001	$\log_{10}()$	.70
**HCT** (daily minimum)	34/49	.004	$()^2$	.08

* Based on Anderson-Darling normality test;

[L] Total days less than 30 (due to early death or a transfer) were identified as outliers and were excluded from the normality test; HCT: Hematocrit percentage in blood;

Table S2. Group-wise normality test and transformation needed

Variables	N (available)			Normality (*P-value)			[&]Transformation function	Normality after transformation (P-value)		
**IVH status** (**No**/**M**ild/**S**evere)	**No**	**M**	**S**	**No**	**M**	**S**		**No**	**M**	**S**
**SBF$_i$** (median level/day, e-8*cm^2/s)	27	9	13	.36	.21	.41	-	-	-	-
**CBF$_i$** (median level/day, e-8*cm^2/s)	27	9	13	.05	.09	<.01	$\log_{10}()$	.70	.27	.07
**CV$_{SBFi}$** (Coefficient of Variation median/day using 5-minute sliding window)	27	9	13	.35	.28	<.01	logit()	.07	.27	.14
**CV$_{CBFi}$** (5-minute sliding window median/day)	27	9	13	.59	.15	.80	-	-	-	-
**CV$_{CBFi_20}$** (20-minute sliding window median/day)	27	9	13	.39	.75	.37	-	-	-	-
**CV$_{CBFi_40}$** (40-minute sliding window median/day)	27	9	13	.054	.71	.07	-	-	-	-
**RiSC** (average coefficient/day)	27	9	13	.64	.23	.25	-	-	-	-
**T$_{RiSC}$** (average seconds/day)	27	9	13	.08	.01	.68	$\sqrt{()}$	.19	.11	.60
**MAP** (median level/day, mmHg)	11	2	4	.35	n/a	.78	-	-	-	-
**CV$_{MAP}$** (5-minute sliding window median/day)	11	2	4	.16	n/a	.59	-	-	-	-
**Pressure passiveness** (average correlation coefficient/day)	11	2	4	.54	n/a	.72	-	-	-	-
**T$_{Pressure\ passiveness}$** (average seconds/day)	11	2	4	.63	n/a	.08	-	-	-	-

[&] The same transform was applied to each group when at least one group did not pass the normality test;
* Based on Anderson-Darling normality test;
No: No-IVH group; M: Mild-IVH group; S: Severe-IVH group;
n/a: Not applicable due to small sample size (N=2).

Table S3. Multivariate, linear mixed modeling results (*P* values) associated with IVH groups of no-IVH, mild-IVH, and severe-IVH (daily repeated measures were set as 'random' effect)

		As a factor		As both a factor and interaction			
Outcome	**Covariate tested**	***IVH effect***	**Covariate effect**	***IVH effect***	**Covariate effect**	**Interaction term**	**Note**
CV_{CBFi}	Gestational age	*.96*	.67	.91	.69	.26	
	Birth weight	*.96*	.14	*.59*	.29	.99	
	Apgar 1 min	*.99*	.62	.55	.26	.066	Higher Apgar much lower CV in mild-IVH only
	Apgar 5 min	*.60*	.04	Ns	Ns	Ns	Higher CV with higher Apgar
	HCT^2 (daily min)	*.58*	.98	.45	.39	.04	Higher HCT was higher CV, but in opposite direction in severe-IVH
	PDA	*.95*	.96	LDF	LDF	LDF	
	Time of IVH found by HUS	*.83*	.56	*.96*	.97	.55	Tested only between mild vs severe IVH groups
	MAP	*.22*	.20	Ns	Ns	Ns	
	CV_{MAP}	*.56*	<.001	Ns	Ns	Ns	Strong positive relationship
	Caffeine y/n	*.86*	.06	*.55*	.09	.39	Smaller CV with Caffeine
	INDO y/n	*.95*	.19	LDF	LDF	LDF	
	Inotropes y/n	*.86*	.15	LDF	LDF	LDF	
	ACE y/n	*.90*	.17	LDF	LDF	LDF	
	Ventilation	*.97*	<.01	*.92*	.04	.61	Higher cv when non-invasive
RiSC	Gestational age	*.16*	.52	*.18*	.53	.35	
	Birth weight	*.03*	.03	*.13*	.18	.84	Larger BW more RiSC
	Apgar 1 min	*.14*	.41	*.99*	.40	.25	
	Apgar 5 min	*.12*	.34	*.14*	.63	.63	
	HCT^2 (daily minimum)	*.15*	.19	Ns	Ns	Ns	
	PDA	*.11*	.24	LDF	LDF	LDF	
	Time of IVH found by HUS	*.19*	.61	*.24*	.95	.61	Tested only between mild vs severe IVH groups
	MAP	*.52*	.87	*.77*	.75	.44	
	CV_{MAP}	*.42*	.10	Ns	Ns	Ns	
	Caffeine y/n	*.20*	.34	*.14*	.68	.58	
	INDO y/n	*.17*	.50	LDF	LDF	LDF	
	Inotropes y/n	*.23*	.27	LDF	LDF	LDF	
	ACE y/n	*.17*	.96	LDF	LDF	LDF	
	Ventilation	*.10*	.12	*.12*	.15	.96	Higher RiSC when non-invasive

LDF: Lost degree of freedom; Ns: Not significant at all (Determined by JMP software);
PDA: Patent Ductus Arteriosus;
INDO: Indomethacin; ACE: Acetaminophen

Apgar score at 5 minutes vs RiSC by IVH groups

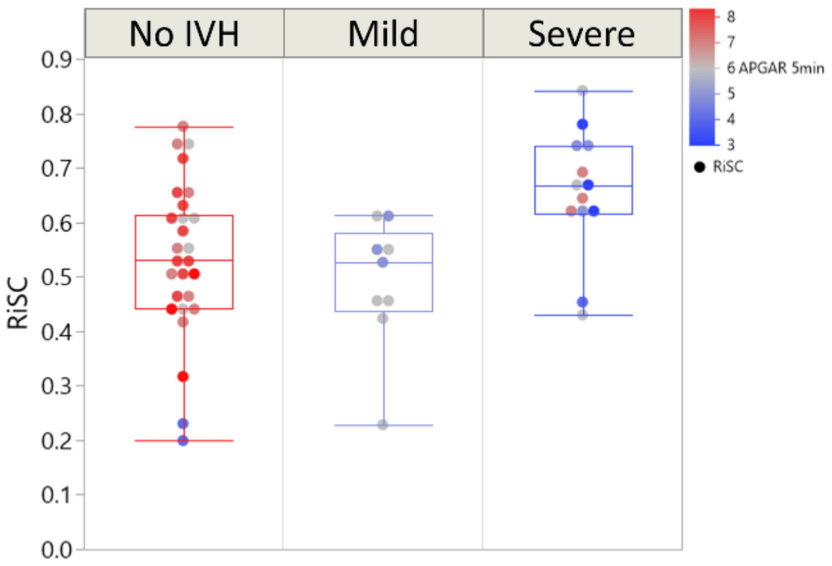

Figure S3. The large variability of RiSC in the No-IVH group may be due to more well-developed, healthier preterm (higher Apgar scores) infants that were better able to sustain and tolerate the pressure passive events and dysregulation of CBF compared to other infants who were diagnosed with IVH under the same factors.

Possible trends in RiSC to the time of IVH onset

To see whether the daily CAR indices could track the development of IVH, we plotted the RiSC over the time distance from [time of the DCS measurement]-to-[time of IVH diagnosed by HUS], as shown in Figure S4. Based on all the IVH cases, there was a strong correlation between the RiSC increase and time of IVH onset (P=.03, R^2=.20, not shown). However, we further stratified the IVH cases into mild- and severe-IVH groups, in order to address the unbalanced observation seen in the severe-IVH cases. This was largely due to the narrow window of opportunity for measuring before the IVH occurred, as the onset of IVH was 3 times earlier in the severe-IVH group compared to the mild group, in addition to the inherent difficulty of recruitment immediately at the time of birth (Table 1, Average time to severe IVH, and the time of the 1st DCS measurements were 45 and 30 HoL, respectively).

As a result, the mild-IVH group showed a weakly positive correlation between RiSC and the time to IVH discovery (P=.20, R^2=.22, a linear fit without accounting for repeated measures in each subject, solid green line in Figure S4-a). In the severe-IVH group, there was no significant correlation between RiSC and the time to IVH discovery (P=.80, R^2=.01, red dashed line in Figure S4-a). And, no correlation was found between RiSC and the hours of life in any group (Figure S4-b). These trends were similar when using T_{RiSC} as an outcome variable of the model. This showed potential utility in tracking the pathogenesis of the cerebral hemorrhage, yet more detailed and sophisticated time analyses are needed with a larger sample size. Meanwhile, we did not find a consistent and notable association between CBF fluctuation indices using a 5-, 20-, or 40- minute sliding window and the time to IVH onset (Not shown).

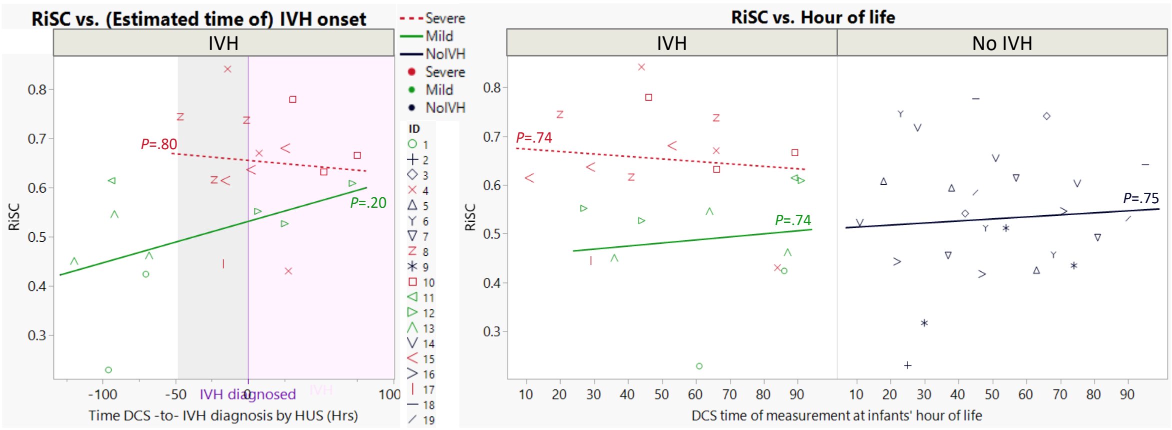

(a) (b)

Figure S4. RiSC was positively trended towards the onset of IVH, although the sample size is small and unbalanced in the severe IVH cases due to their early onset of IVH shortly after birth. Each point was based on one day of measurement. The group of the same symbol represents the repeated measures in each subject. (a) RiSC was trended towards the onset of IVH in the mild-IVH case, while not enough observations were available to draw conclusions in the severe-IVH cases. (b) Meanwhile, there were no specific trends in the RiSC over the infants' hours of life. Red= severe-IVH; Green= mild-IVH; Black= no-IVH.

Sensitivity analysis

Sensitivity analyses showed that RiSC values were not sensitive to using different window sizes (of 2, 3, 5, and 10 minutes), but were sensitive to added noise (Figure S5). To increase the robustness of this method under a noisy environment, it is possible to obtain the statistical significance of each correlation coefficient against a bootstrapped null distribution based on the remainder of the measurement [37]. This can help normalize the index to the baseline noise level as well as sort out any spurious correlations that could occur by chance. Nevertheless, multiple hours and days of spontaneous measurements used in our study helped obtain more reliable indices of possible dysfunction in CAR.

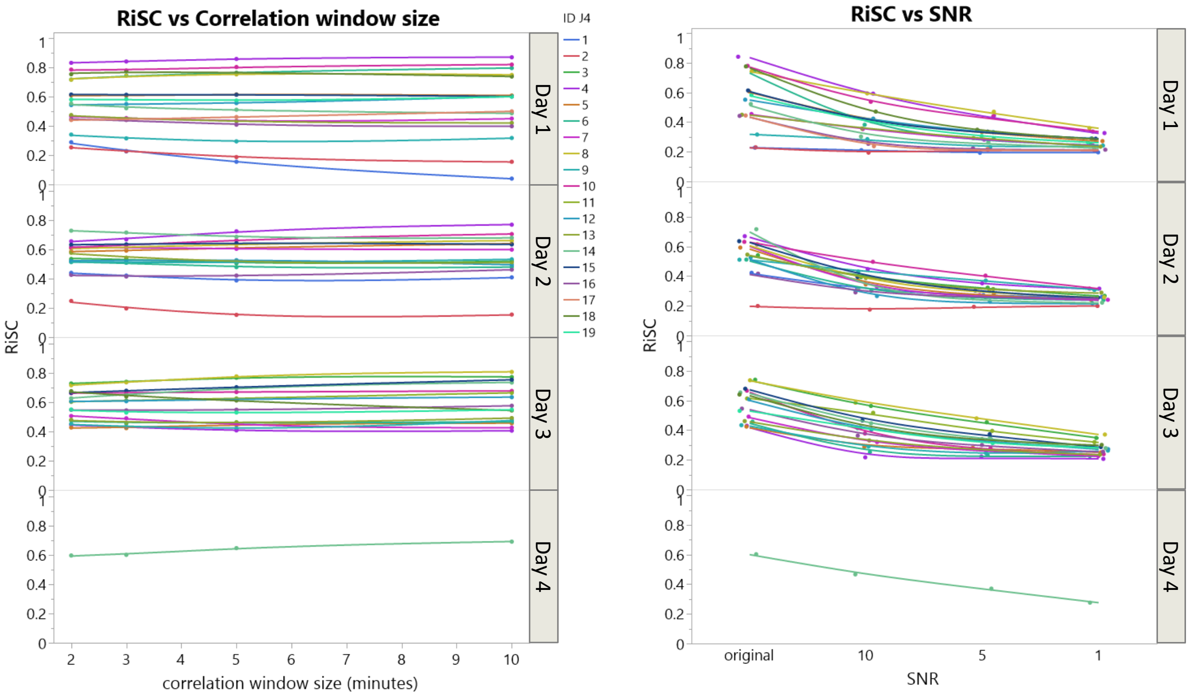

Figure S5. RiSC sensitivity: across different correlation window size (left) and injected noise level (right) suggested the RiSC indices were not sensitive to window size, while they were sensitive to added noise. Each point represents a daily measurement.
